# Electrochemical Voltammogram Recording for Identifying Varieties of Ornamental Plants

**DOI:** 10.3390/mi11110967

**Published:** 2020-10-29

**Authors:** Rutong Yang, Boyuan Fan, Shu’an Wang, Linfang Li, Ya Li, Sumei Li, Yuhong Zheng, Li Fu, Cheng-Te Lin

**Affiliations:** 1 Jiangsu Key Laboratory for the Research and Utilization of Plant Resources, Institute of Botany, Jiangsu Province and Chinese Academy of Sciences, Nanjing 210014, China; yangrt2000@cnbg.net (R.Y.); lzwandwsa@163.com (S.W.); lilinfangqq@163.com (L.L.); smli321@163.com (S.L.); zhengyuhong@cnbg.net (Y.Z.); 2 College of Materials and Environmental Engineering, Hangzhou Dianzi University, Hangzhou 310018, China; 191200023@hdu.edu.cn; 3 Key Laboratory of Marine Materials and Related Technologies, Zhejiang Key Laboratory of Marine Materials and Protective Technologies, Ningbo Institute of Materials Technology and Engineering (NIMTE), Chinese Academy of Sciences, Ningbo 315201, China; linzhengde@nimte.ac.cn; 4 Center of Materials Science and Optoelectronics Engineering, University of Chinese Academy of Sciences, Beijing 100049, China

**Keywords:** electrochemical sensor, voltammograms, variety identification, pattern recognition, *Clematis* spp.

## Abstract

An electrochemical voltammogram recording method for plant variety identification is proposed. Electrochemical voltammograms of Vistula, Andromeda, Danuta, Armandii ‘Apple Blossom,’ Proteus, Hagley Hybrid, Violet Elizabeth, Kiri Te Kanawa, Regina, and Veronica’s Choice were recorded using leaf extracts with two solvents under buffer solutions. The voltametric data recorded under different conditions were derived as scatter plots, 2D density patterns, and hot maps for variety identification. In addition, the voltametric data were further used for genetic relationship studies. The dendrogram deduced from the voltammograms was used as evidence for relationship study. The dendrogram deduced from voltametric data suggested the Andromeda, Danuta, Proteus, Regina, and Hagley Hybrid were closely related, while Violet Elizabeth and Veronica’s Choice were closely related. In addition, Vistula and Armandii ‘Apple Blossom’ could be considered outliers among the varieties.

## 1. Introduction

The typical morphological features of ornamental plants are specific, visually visible external features. They can directly reflect the evolutionary course and genetic relationship of plants. Therefore, they can be used as the main bases for variety identification and classification. Though comparative morphological methods are relatively simple and intuitive [1,2], plant morphology is affected not only by environmental conditions but also by gene expression [3,4]. Therefore, the accurate definition of stable, inherited morphological traits is a prerequisite for comparative morphological identification and classification. The cytological method has the characteristics of strong stability, but it can only can provide limited information [5,6,7]. It is difficult to obtain a clear classification result with this method when performing karyotype analysis between varieties [8,9,10,11]. Therefore, a large sample size is a prerequisite for cytological identification and classification. The morphological characteristics of pollen are mainly controlled by genes and have strong genetic stability. However, at present, most researchers only use scanning electron microscopy to observe the external form of pollen, and they cannot observe important classification information [12,13,14], which makes identification and classification results deviate from the results obtained by other methods. Quantitative classification methods can comprehensively analyze a large number of biological traits [15,16]. However, in the process of quantitative classification, many steps such as selected trait types, coding rules, data standardization methods, and cluster analysis methods are very subjective, which makes it difficult to compare the results with other methods [17]. Isozymes are the direct products of gene expression. The amount of enzyme bands and changes in mobility are largely determined by structural genes [18,19,20]. Therefore, the existence and expression of genes can be represented according to the isozyme phenotype. However, the polymorphism of plant isozymes is low, resulting in a poor stability. At the same time, this polymorphism is susceptible to environmental factors, sampling locations, and plant development stages [21,22]. Molecular markers have the advantages of a high stability, good repeatability, and wide distribution in the genome [23,24]. However, they are affected by factors such as sample size, the number of markers, and polymorphism. It can be seen that any classification method has its limitations. It is difficult to ensure the reliability of results when using a particular method for variety identification.

In this work, we propose an electrochemical approach for variety identification. Electrochemical methods have been widely used for analytical purpose for sensing [25,26,27,28,29,30,31,32,33,34]. The voltametric scanning of an extract of plant leaves was performed using a glassy carbon electrode. Ten varieties of *Clematis* were deliberately selected as investigation targets [35,36,37]. We recorded the voltammograms of *Clematis* under different conditions and found that different extraction solvents and buffer solutions presented different profiles. Integrating these voltammograms can be used to quickly identify varieties of *Clematis*. In addition, we further propose a new pattern recognition method in this work. The dendrogram deduced from the voltammograms of 10 varieties of *Clematis* gave a persuasive genetic relationship result with breeding records.

## 2. Materials and Methods

Leaves of Vistula, Andromeda, Danuta, Armandii ‘Apple Blossom,’ Proteus, Hagley Hybrid, Violet Elizabeth, Kiri Te Kanawa, Regina, and Veronica’s Choice were collected from Nanjing Botanical Garden Memorial Sun Yat-Sen (Nanjing, China) in April 2020. Table 1 shows all the important information for these varieties. Healthy leaves of each variety were carefully collected and stored at −20 °C before analysis. KH_2_PO_4_, Na_2_HPO_4_, sodium acetate, and acetic acid were purchased from Macklin Co., Ltd. All other chemicals were analytical-grade reagents and were used without further purification. The reference electrode (Ag/AgCl), counter electrode (Pt wire), and working electrode (glassy carbon electrode (GCE), 3 mm in diameter) were all purchased from Gaoshi Ruilian Co. Ltd. (Wuhan, China). Milli-Q water (18.2 MΩ/cm) was used throughout the experiments.

Water and ethanol were directly used as solvents for plant leaf extract preparation. As is typical, 10 mL of water or ethanol were added into 2 g of chopped plant leaf with 1 min of grinding, and then a 0.1 M acetate buffer (ABS, pH 4.5) or a 0.1 M phosphate buffer (PBS, pH 7) was added for 3 min of sonication. For electrochemical fingerprint recording, a GCE was polished with an alumina–water slurry and rinsed with ethanol and water. Then, a three-electrode system was inserted into the beaker for electrochemical fingerprint recording. All electrochemical fingerprints were recorded using a CHI760E working station. Differential pulse voltammetry was used to record the electrochemical fingerprints of all plant tissue between −0.1 and 1.5 V with a pulse amplitude of 50 mV, a pulse width of 0.05 s, and a pulse period of 0.5 s.

For scatter plot, 2D density plot, and hot map generation, the normalized current values recorded after two solvent extractions were used as the *x* and *y* axes. All figures were constructed using Origin2020.

## 3. Results

**Figure 1:**
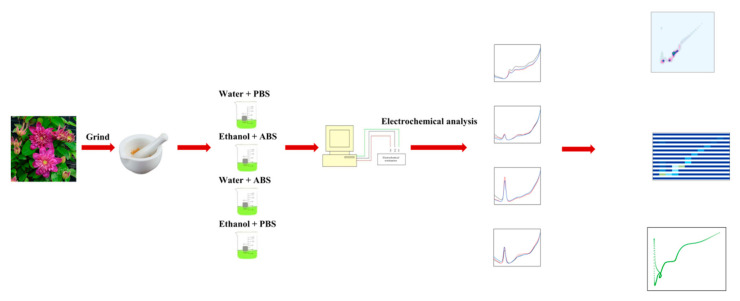
Schematic diagram of the steps involved in the electrochemical approach applied to the of *Clematis* variety identification.

Figure 1 shows a schematic diagram of the voltametric recording of *Clematis*. The leaf extraction process was conducted before voltametric recording. Water and ethanol were used as solvents for extracting the electro-active molecules from the *Clematis* leaf. Then, either the ABS or the PBS was added as electrolyte to provide sufficient ions for an electrochemical reaction. The electrochemical reaction was taken at the surface of the GCE using a differential pulse voltammetry (DPV) scan. The recorded voltammograms of *Clematis* were then submitted for pattern generation. Scatter plots and 2D density patterns have previously been used for plant identification [38,39,40,41,42,43,44,45]. In this work, we further proposed a hot map pattern for variety identification.

**Figure 2:**
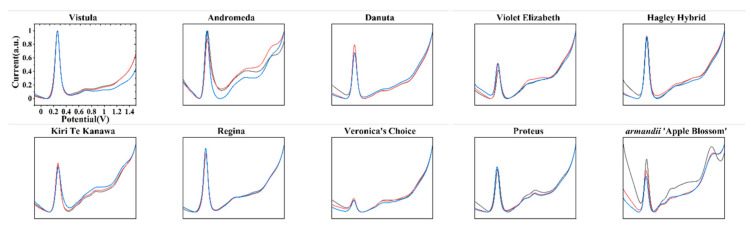
Differential pulse voltammetry (DPV) curves of Vistula, Andromeda, Danuta, Armandii ‘Apple Blossom,’ Proteus, Hagley Hybrid, Violet Elizabeth, Kiri Te Kanawa, Regina, and Veronica’s Choice recorded in a 0.1 M phosphate buffer (PBS) after water extraction.

Figure 2 shows the DPV curves of samples obtained under the 0.1 M PBS after the water extraction process. It can be seen that each variety exhibited several peaks between −0.1 and 1.5 V. In addition, three scans showed an acceptable reproducibility, suggesting that the used method is feasible. These oxidation peaks can be ascribed to molecules in plant tissue such as flavanols [46,47,48,49], phenolic acids [50,51,52,53], procyanidins [54,55,56], alkaloids [57,58,59], and pigments [60,61,62]. In plant extraction, ethanol is more suitable for the extraction of volatile oil, organic acids, resins, alkaloids, polyphenols, and flavonoids. The identification of these compounds is a big challenge because no separation process was able to be used for the sample. However, the total electrochemical profile could be considered to show the overall information of the electro-active compounds in the plant tissue. Therefore, it shows potential for the quick identification of plants.

**Figure 3:**
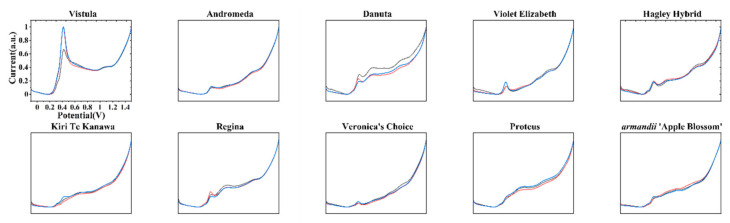
DPV curves of Vistula, Andromeda, Danuta, Armandii ‘Apple Blossom,’ Proteus, Hagley Hybrid, Violet Elizabeth, Kiri Te Kanawa, Regina, and Veronica’s Choice recorded in a 0.1 M acetate buffer (ABS) after ethanol extraction.

Figure 3 shows the DPV curves of samples obtained under the 0.1 M ABS after ethanol extraction. Each variety of *Clematis* also showed oxidation peaks during the scan. Differences between these curves and the curves obtained in Figure 2 can be observed due to the different electro-active compounds that participated in the electrochemical reaction.

**Figure 4:**
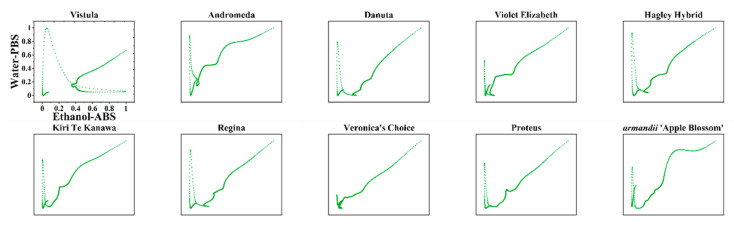
Scatter plots of Vistula, Andromeda, Danuta, Armandii ‘Apple Blossom,’ Proteus, Hagley Hybrid, Violet Elizabeth, Kiri Te Kanawa, Regina, and Veronica’s Choice using data recorded from the 0.1 M PBS after water extraction vs. the 0.1 M ABS after ethanol extraction.

The direct recognition of varieties using DPV curves is quite hard because the DPV curves of some varieties showed similar features, like with the voltammograms of Danuta and Regina. In addition, the DPV curves of Kiri Te Kanawa and Proteus also exhibited very similar profiles. In order to quickly identify these varieties of *Clematis*, the DPV curves recorded under different conditions could be used for generating different patterns. Figure 4 shows the scatter plots of all 10 varieties using data recorded from the 0.1 M PBS after water extraction against the 0.1 M ABS after ethanol extraction. The increasing the dimension of data obviously improved the difference between samples. For example, the difference between Kiri Te Kanawa and Proteus was more obvious in the scatter plots than the difference observed between the DPV curves.

The use of scatter plots for variety identification still has certain limitations. Since each data point has the same weight, it is difficult to intuitively find differences between varieties from scatter plots. In order to highlight the key points of data, we used the data recorded from the 0.1 M PBS after water extraction and the 0.1 M ABS after ethanol extraction to make a 2D density plot.

**Figure 5:**
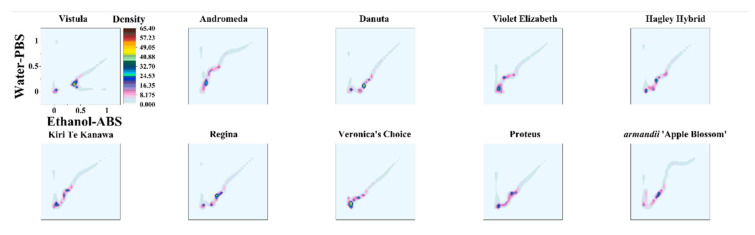
2D density plots of Vistula, Andromeda, Danuta, Armandii ‘Apple Blossom,’ Proteus, Hagley Hybrid, Violet Elizabeth, Kiri Te Kanawa, Regina, and Veronica’s Choice using data recorded from the 0.1 M PBS after water extraction vs. the 0.1 M ABS after ethanol extraction.

In the 2D density plot, the area with more data points is highlighted. We can distinguish different varieties by simply locating the highlighted area. Figure 5 shows the 2D density plots of Vistula, Andromeda, Danuta, Armandii ‘Apple Blossom,’ Proteus, Hagley Hybrid, Violet Elizabeth, Kiri Te Kanawa, Regina, and Veronica’s Choice. Most varieties can be identified by locating their highlighted area, but there are still some varieties that have similar patterns.

**Figure 6:**
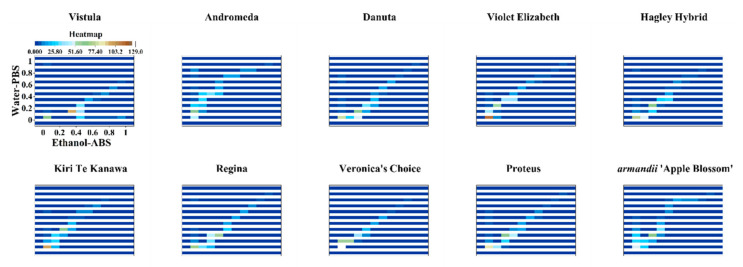
Hot maps of Vistula, Andromeda, Danuta, Armandii ‘Apple Blossom,’ Proteus, Hagley Hybrid, Violet Elizabeth, Kiri Te Kanawa, Regina, and Veronica’s Choice using data recorded from the 0.1 M PBS after water extraction vs. the 0.1 M ABS after ethanol extraction.

Therefore, we further proposed a new pattern identification approach. The data points of the DPV curves recorded from the 0.1 M PBS after water extraction and the 0.1 M ABS after ethanol extraction were used to construct a hot map. Figure 6 shows the hot maps of Vistula, Andromeda, Danuta, Armandii ‘Apple Blossom,’ Proteus, Hagley Hybrid, Violet Elizabeth, Kiri Te Kanawa, Regina, and Veronica’s Choice. It can be seen that the hot map divided all data into many small cubes. Each cube represents the hot degree of the data compared with all of the data. This identification method makes it much easier to locate hot zones and perform quantitative analysis than a 2D density plot. For example, Proteus and Regina had very similar 2D density plots, as seen in Figure 5. However, we could easily recognize them by the six yellow and red-purple cubes that appear at the beginning of their hot maps.

**Figure 7:**
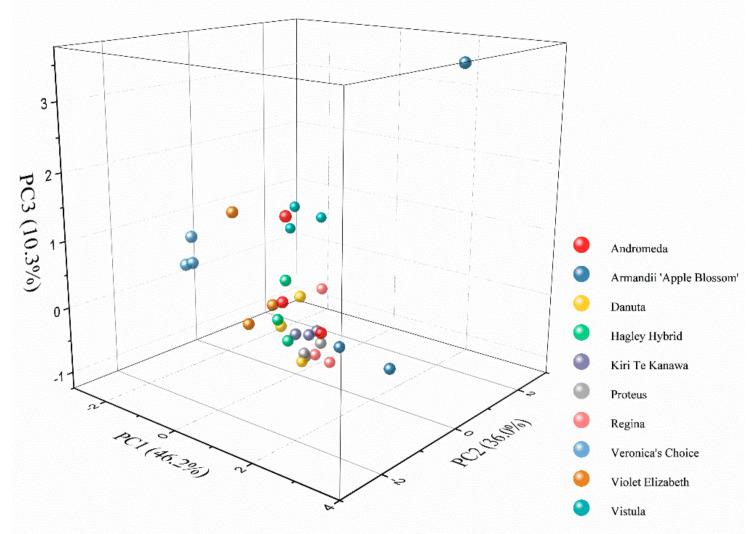
3D principal component analysis (PCA) analysis of Vistula, Andromeda, Danuta, Armandii ‘Apple Blossom,’ Proteus, Hagley Hybrid, Violet Elizabeth, Kiri Te Kanawa, Regina, and Veronica’s Choice using normalized current recorded after two solvent extractions under two buffer solution conditions.

In order to further testing the difference of electrochemical profile between the species, a principal component analysis (PCA) analysis was carried out. All four sets of data (0.1 M PBS after water extraction, 0.1 M PBS after ethanol extraction, 0.1 M ABS after ethanol extraction, and 0.1 M ABS after water extraction) were submitted for the PCA analysis. As shown in Figure 7, three samples of each variety showed very close locations, suggesting the good reproducibility of the proposed method. On the other hand, the three factors extracted within the voltametric data could reach more than 90% of interpretative capability, suggesting that there were significant differences in the electrochemical profiles of the varieties. The 3D PCA grouping result showed that Danuta, Violet Elizabeth, Hagley Hybrid, and Kiri Te Kanawa were grouped in a close spatial position. In addition, Armandii ‘Apple Blossom’ and Vistula could be considered outliers among the varieties. The results suggest the obvious differences in electro-active compounds among varieties, reflecting that there may also be significant differences at the gene level.

**Figure 8:**
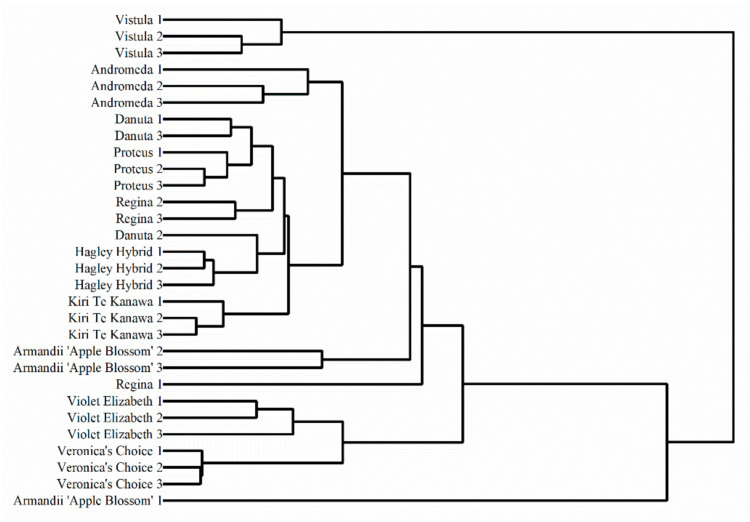
Dendrogram of Vistula, Andromeda, Danuta, Armandii ‘Apple Blossom,’ Proteus, Hagley Hybrid, Violet Elizabeth, Kiri Te Kanawa, Regina, and Veronica’s Choice based on the voltammograms recorded after two solvent extractions under two buffer solution conditions.

Since the voltammograms of the varieties were positively correlated with the distribution and amount of electro-active compounds, we attempted to use the electrochemical data for dendrogram analysis. Figure 8 shows the dendrogram of Vistula, Andromeda, Danuta, Armandii ‘Apple Blossom,’ Proteus, Hagley Hybrid, Violet Elizabeth, Kiri Te Kanawa, Regina, and Veronica’s Choice deduced from the voltammograms recorded after two solvent extractions under two buffer solution conditions. The phylogenetic tree was divided into two main principal clades. The first clade consisted of the varieties of Andromeda, Danuta, Proteus, Regina, and Hagley Hybrid. The second clade consisted of the varieties of Violet Elizabeth and Veronica’s Choice. In addition, Vistula and Armandii ‘Apple Blossom’ could be considered outliers among the varieties. Based on the information in Table 1, Vistula is the only variety in late large-flowered group among them, while the Armandii ‘Apple Blossom’ is the only variety in the Armandii group. These results suggested that the difference observed in the voltammograms may be linked to the distance between the genetics.

## 4. Conclusions

In conclusion, the voltammograms of Vistula, Andromeda, Danuta, Armandii ‘Apple Blossom,’ Proteus, Hagley Hybrid, Violet Elizabeth, Kiri Te Kanawa, Regina, and Veronica’s Choice were recorded by a GCE using plant leaf extracts. Based on the recorded voltammograms, these varieties could be effectively identified using pattern recognition. A hot map was used for the first time for plant identification based on voltametric data. In addition, voltammograms were used for phylogenetic analysis. The dendrogram deduced from voltammograms suggests that Andromeda, Danuta, Proteus, Regina, and Hagley Hybrid are closely related, while Violet Elizabeth and Veronica’s Choice are closely related. In addition, Vistula and Armandii ‘Apple Blossom’ can be considered outliers among the varieties.

## Figures and Tables

**Table 1:** Information of Vistula, Andromeda, Danuta, Armandii ‘Apple Blossom,’ Proteus, Hagley Hybrid, Violet Elizabeth, Kiri Te Kanawa, Regina, and Veronica’s Choice.

Variety Name	Group	Approximate Height	Country of Origin	Parentage
Vistula	Early large-flowered group	2.0–3.0 m	Poland	Unknown
Andromeda	Early large-flowered group	2.0–4.0 m	United Kingdom	Seedling of ‘General Sikorski’
Danuta	-	2.0–2.5 m	Poland	Unknown
Armandii ‘Apple Blossom’	Armandii group	5.0–7.0 m	China	Unknown
Proteus	Early large-flowered group	2.5–3.0 m	United Kingdom	C. viticella ‘Grandiflora’ x ‘Fortunei
Hagley Hybrid	Late large-flowered group	2.0–3.0 m	United Kingdom	Unknown
Violet Elizabeth	Early large-flowered group	2.0–3.5 m	United Kingdom	‘Vyvyan Pennell’ x ‘Mrs. Spencer
Kiri Te Kanawa	Early large-flowered group	2.0–3.0 m	United Kingdom	‘Beauty of Worcester’ x ‘Chalcedony’
Regina	Early large-flowered group	1.5–2.0 m	Poland	Unknown
Veronica’s Choice	Early large-flowered group	2.5–3.0 m	United Kingdom	‘Vyvyan Pennell’ x ‘Percy Lake’
